# Physicochemical, Structural, Molecular, and Thermal Characterization of *Fucus vesiculosus* Extract-Based Nanofibrous Mats

**DOI:** 10.1021/acsomega.5c09347

**Published:** 2026-01-12

**Authors:** Fatih Bildik

**Affiliations:** 600680 Istanbul Technical University, Faculty of Chemical and Metallurgical Engineering, Department of Food Engineering, Istanbul 34469, Türkiye

## Abstract

Ratios of 3:1 and 4:1 *Fucus vesiculosus*:zein (FV:Z) containing nanofibrous mats were designed for the first time by the electrospinning method. According to the results, electrical conductivity, surface tension, viscosity, and density are proportional to each other. Scanning electron microscopy (SEM) images depicted morphology, revealing the presence of homogeneous nanostructures. Changing the polymer ratios altered the fiber diameter, specific surface area, pore volume, and pore size. Average values of hydroxycinnamic (21.5 μgmg^–1^), quercetin (19.8 μgmg^–1^), and caffeic (16.23 μgmg^–1^) were determined to be the highest phenolic compounds in the HPLC chromatogram, serving as an important source of bioactive ingredients for food applications. Total IC50 values of FV:Z 3:1 and FV:Z 4:1 mats were 46.37 μgml^–1^ and 46.90 μgml^–1^, respectively. Total phenolic content (TPC) is also presented at higher levels in all of the ABTS and DPPH assays. Determination of the H atom in the −OH group and the carbonyl group of a carboxylic acid group of phenolic acid in the FTIR spectrum was consistent with the ABTS and DPPH results. In the FTIR spectra for nanofibrous mats, vibrations shifted toward smaller wavenumbers with stronger ionic interaction compared to the FV extract. Slightly shifted and increased peaks were observed in the spectra, confirming successful interaction between Fucus and zein. Functional groups (NH_2_, COOH, and OH) allow intermolecular interactions such as H bonding. π–π stacking, π alkyly, and sulfur-containing products are detected. TGA and DSC results showed that the thermal stability of nanoencapsulated FV bioactive compounds strengthened at higher temperatures. Differences in XRD crystal patterns, such as changes in the intensities of diffraction peaks, were also recognized between samples. Overall, stability and bioactivity improved with the formation of inter/intramolecular associations. Results indicated that nanofibrous mats have potential in the nanoencapsulation and delivery of bioactives.

## Graphical abstract

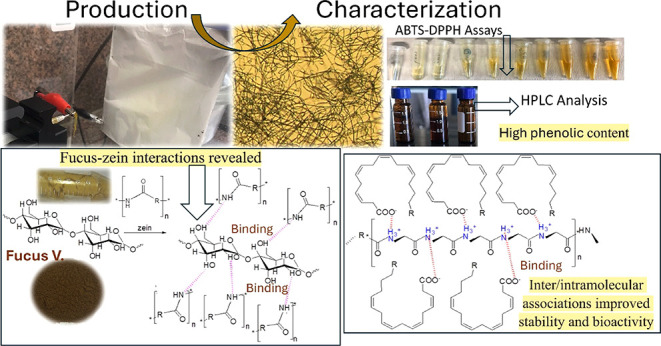

## 1 Introduction

Algae are multicellular and unicellular microorganisms that grow in diverse environmental conditions. They are rich sources of protein, polysaccharides, dietary fiber, pigments, vitamins, and minerals. Microalgae-based products are nontoxic for humans; therefore, their utilization has attracted increasing attention in recent years. Among these, the fucan family is an important source of high-value bioactive molecules such as polyphenols. *Fucus vesiculosus* (FV) is one of the more common species of the genus Fucus that grows in moderately cold water and at depths of 0.5–5 m in desalinated bays. FV contains polyphenols, flavonoids, fucoidan, essential fatty acids, minerals, and vitamins. Phlorotannin extracts of this genus have also been reported for their promising antioxidant, antiinflammatory, and antitumor activities.^1–3^ Phlorotannins are polyphenolic compounds with biological activity in brown microalgae, with their concentration reaching up to 30% of the biomass of Fucus species.^1,2^ Circuncisao et al.^4^ reported the ability of FV to accumulate up to 15% minerals. FV cell walls contain fucoidan polysaccharides, which are composed of different amounts of xylose, sulfate, galactose, and uronic acids.^5^ These soluble polysaccharide fiber groups (laminarans and alginates) are renowned for exhibiting biological properties, including anticoagulant and antioxidant activities. It has also been reported that Fucus microalgae species provide advantages in biotechnology and commercial fields as new protein sources.^6,7^

Brown algae applications have been more appealing to researchers during the last decades.^8^ Widespread specimens of Fucus are rich sources of protein (70%) and nonprotein compounds, such as dietary fiber and amino acids. Therefore, Fucus species are used in food ingredients, pharmaceutical industries, animal feed, and cosmetics. Sulfated polysaccharides and essential fatty acids, which are major compounds, also provide benefits for health.^6,8^ Fucoidan was extracted from brown algae using ultrasound, microwave, and a sequential combination of ultrasound and microwave methods to compare their effects with the conventional method.^9–12^ It can also be used for nonthermal nanotechnological approaches.

Electrospun nanofibers can enhance efficiency, solubility of encapsulated bioactive compounds, bioavailability, and stability.^13,14^ Recently, antioxidant mats have been fabricated using microalgal compounds with biopolymers such as gelatin^15–17^ or polyvinyl alcohol.^18^ Although *Fucus vesiculosus* has numerous promising properties, its electrospinning presents challenges due to its low viscoelasticity and solubility. It has been reported that other natural polymers, such as chitosan, cellulose, and sodium alginate, can facilitate its electrospinning for the formation of electrospun nanofibers.^19^ However, mainly acids or surface-active agents have been used for microalga-based nanofibers, or their ratio remains low for other polymers. Natural polymers ca be electrospun with the assistance of additives. Researchers have studied the possibility of additive-free electrospinning of natural polymers. For instance, Kopp et al.^20^ controlled the viscosity of the spinning silk fibroin solution by partial evaporation of the initial dissolution.

The branched structure and negatively charged SO_4_^2–^ groups of fucoidan contribute to stability. Fucoidan enables high encapsulation efficiencies for various bioactives due to its capacity to interact with hydrophobic molecules through covalent and noncovalent interactions.^21^ It forms gels with other proteins, enhancing intermolecular interactions. Carrageenan, alginate, or gelatin can lead to gelation through entanglement and ionic and H bonding with fucoidan. In this sense, zein is also a promising globular protein used in the food industry due to its biocompatibility, biodegradability, and nontoxicity. Qiufan et al.^22^ reported that the use of bioactive-loaded zein mats preserves the polyphenol contents of *Spirulina platensis* and improves physicochemical properties. Considering the above-mentioned studies, the application of sulfated polysaccharides in protein–polysaccharide systems aims to provide a reference for their use in the encapsulation and delivery of hydrophobic active substances in algae extracts. Therefore, zein biopolymer at a low ratio is utilized in the electrospinning process with FV extracts to obtain electrospun mats, which are then characterized in terms of their physicochemical, morphological, molecular, and thermal properties.

## 2 Material and Methods

### 2.1 Material

Dried FV extract and zein were purchased from Gökçek ŞİFA Laboratory Industry and Trade Co. Ltd. and Sigma-Aldrich, respectively. HPLC standards, ABTS, and DPPH assay standards were obtained from Sigma.

### 2.2 Solution Preparation, Properties Determination, and Electrospinning Process

Solutions were prepared by dissolving FV extracts and zein (at ratios of 3:1 and 4:1) in 70/30 ethanol/water mixtures. Thus, total biopolymer concentrations were 40% and 50% for 3:1 and 4:1 FV:Z mixed with solvent, respectively. A pH meter (Hanna), density meter, surface tension meter (Dataphysics), electrical conductivity meter (WTW), and viscosity meter (Brookfield) were utilized, with three repetitions, to determine the electrospinnable solution properties. The Haake Rheostress Rheometer was used to determine dynamic viscosity. The syringe was placed on a horizontal pump system (KD-Scientific Legato) for the electrospinning process. Pretrials were performed at 0.5, 0.8, and 1.2 mL/h flow rates at room temperature. The optimum conditions were achieved at 0.8 mL/h flow rate, 15 kV constant applied voltage by using a high-voltage power supply (Spellman, SL Series), and a 15 cm tip-to-metal collector distance.

### 2.3 Characterization of Electrospun Nanofibrous Mats

#### 2.3.1 Diameter, BET Isotherm, and Gibbs Energy Calculations

SEM (Leitz, AMR 1000, Germany) was used to examine nanofibrous mats’ structures. ImageJ software was used to find the average diameters by calculating the sum of 100 random measurements from 5000x magnification images. The Brunauer–Emmett–Teller Kelvin 1042 Sorptometer was used to determine the surface area and pore size of nanofibrous mats, utilizing Image Metrology Software. The method application and calculations were described in a previous study by Bildik et al.^23^

The isotherm model assumes a homogeneous distribution of areas on the surface of the adsorbent, the existence of adsorption energy, and the formation of adsorbent multilayers, with the second energy holding the layers of adsorbed molecules. The model also takes into account the solubility of solids in the solvent, saturation concentration, and saturation phenomenon. The relationship between the adsorption capacity and the concentration at equilibrium is described using the isotherm in its linear (eq 1) form;

1 $$\frac{\mathrm{Ce}}{\mathrm{Qe}(\mathrm{Cs}‐\mathrm{Ce})}=\frac{1}{\mathrm{Qs}\mathrm{CBET}}+\frac{(\mathrm{CBET}-1)\left(\frac{\mathrm{Ce}}{\mathrm{Cs}}\right)}{\mathrm{Qs}\mathrm{CBET}}$$

where CBET is the BET adsorption isotherm relating to the energy of surface interaction (L mg^–1^); Ce: equilibrium concentration (mg L^–1^); Cs: adsorbate monolayer saturation concentration (mg L^–1^); Qe: the amount of adsorbate in the adsorbent at equilibrium (mg g^–1^); and Qs: theoretical isotherm saturation capacity (mg g^–1^).

The Gibbs experiments were conducted using thermodynamic parameters as stated by Chakraborty et al.^24^ Interactions between biopolymer active ingredients and FV polyphenols were determined by the thermodynamic properties of the system. Therefore, the free energy change (Δ*G*), entropy change (Δ*S*), enthalpy (Δ*H*), and the Gibbs–Helmholtz (eq 2) are given as below:

2 $$\begin{array}{l} \Delta G=\Delta H-T\Delta S\\ -RT\ln K=\Delta H-T\Delta S \end{array}$$

where *T* is the experimental absolute temperature, *K* is the binding constant at the corresponding temperature, and *R* is the gas constant [8.314 J­(mol K)^−1^]. The temperature was set as 298 and 303 K.

#### 2.3.2 Fourier Transform Infrared Analysis

FTIR analysis was applied by using a Bruker Fourier transform infrared spectrometer. A total of 37 scans were performed at a resolution of 400–4000 cm^–1^. Spectra were analyzed by using OMNIC 9.0 software. Band distance ranges were measured according to previously published study.^25^ Chemical bond interactions in all figures were generated using ChemDraw 8.0 software.

#### 2.3.3 Evaluation of Antioxidant Activity and Capacity by ABTS and DPPH Assays

The antioxidant activity of the polyphenols (fucoidan and phlorotannins) in FV-based electrospun nanomats was determined by DPPH and ABTS methods. To measure antioxidant activity, the mats were dissolved to provide five different concentrations ranging from 2 to 25 mg mL^–1,^ and total antioxidant capacity values (from 10 to 90 μg ml^–1^) were evaluated.

DPPH radical scavenging activity was measured according to Wang et al.^26^ A 0.1 mL of the sample solution (five different concentrations were prepared for each sample) was mixed with 3.9 mL of DPPH solution (64 μM or 2.5 × 10–2 g L^–1^ in methanol). After incubation for 2 h, the absorbance was measured at a 515 nm wavelength using a spectrophotometer. A calibration curve was used to calculate the remaining concentration of DPPH in the reaction medium. The IC50 value was calculated as the concentration of sample or standard antioxidant (μgmL^–1^) required to scavenge 50% of the DPPH in the reaction mixture. The antiradical activities of the nanofibrous mats toward DPPH radicals were determined as described in the methods by Morsi et al.^27^ and Golshany et al.^12^ Trolox concentrations (0–200 μgmL^–1^) were added to 0.2 mM DPPH solution prepared in 2.8 mL of methanol. The mixture was kept in the dark for 30 min at room temperature, and absorbance was measured at 517 nm. The results were presented using a linear standard curve.

The DPPH scavenging activity was determined by measuring the ability of the mats to scavenge the free radical DPPH.^28^ The control solution consisted of distilled water used instead of water for the sample solution. Results were expressed as the inhibition percentage using (eq 1):

% inhibition of DPPH = (1 – Sample solution absorbance/Control solution absorbance) × 100 (1)

The percentages were calculated after 180 min. Graphs were generated using Origin 2022.

#### 2.3.4 Qualification and Quantification of Polyphenol Components via HPLC

The phenolic acids in FV:Z 4:1 and FV:Z 3:1 nanofibrous mats were analyzed by HPLC (Shimadzu, Japan) equipped with binary pumps, an automatic injector, and a photodiode array detector. For phenolic compounds, the mobile phase consisted of solvent A (acetonitrile) and solvent B (0.75% aqueous formic acid solution). The injection volume was 10 μL, and the flow rate was adjusted to 1 mLmin^–1^. The gradients of the setup are varied by changing the proportion of solvent A to solvent B. The gradient elution was changed from 5% to 50% B at 30th min, from 30% to 70% B at40th min, and from 50% to 100% B at 40th min. Lutein, apigenin, hesperidin, and hydroxycinnamic acid were monitored at 220 nm, while quercetin and quercetin-3-gal were monitored at 306 nm, and caftaric acid, ferulic acid, and chlorogenic acid were monitored at 330 nm.

The standard was prepared at different concentrations (10, 20, 30, 40, and 50 μg ml^–1^). The correlation of standards was 1.00.

The method was validated for LOQ and LOD, accuracy, precision, and linearity, according to ICH guidelines (ICH Harmonized Tripartite Guideline, 2001). To determine linearity, the stock solution of the standard (1 mgml^–1^) was diluted to five different concentrations (10, 20, 30, 40, 50 μgml^–1^). The correlation coefficients of all standards were 0.999.

The HPLC techniques have been used for the determination of tested organic samples because of their practicality and simplicity, allowing efficient analysis in all tested samples.^29–31^ On the other hand, the measurement range for trace amounts of polyphenols is difficult because many more interfering components are present in the tested sample. Because of that, an improved analytical method is an important parameter.^32^ Selectivity: the separation of polyphenols (mixed solution and standard mixture) was completed by gradient methods. No interference was observed in the elution times of the amino acids. Linearity: the commercial 18-compound calibration mixture (std PP, sigma) was dissolved in buffer-methanol solution to achieve a final concentration of 0.5 nmol per 10 μL for each polyphenols. Quantified linearity studies were performed to appraise the analytical measurement range for all polyphenols.^33^ The method was linear within an acceptable processing error of 2% up to 500 μM for each polyphenol studied (90 μM gallic acid and coumaric acid, 100 μM chlorogenic acid, and caffeic acid). Under optimal conditions, the limit of quantification (at a signal-to-noise ratio of 8) and the detection limit (at a signal-to-noise ratio of 5) were calculated, respectively. The LOQ values for polyphenols were in the range of 1.1–2.2 μM, and the LOD values were in the range of 3.2–7.1 μM. The correlation coefficient was ≥ 0.9989, with linearity in the range of 0.5 to 500 μM. The intra-assay coefficient of variation for the polyphenols standard was 4.3–6.7%, and the interassay (day-to-day) coefficient of variation was 0.6–2.9%. To optimize the chromatographic procedure, the eluent concentration at pH 3.1 (0.75% formic acid in water) was determined. Optimization of sample mat preparation: the tag method was successfully applied for the analysis of different concentrations of analytes in a variety of matrices.^34^ Different elution solvents were investigated, and it was shown that the buffer solution (pH: 5.5) could elute all analytes.

#### 2.3.5 Examination of Crystallinity

Nanofibrous mats’ crystalline and amorphous regions were examined by an X-ray diffractometer (Bruker D8 Advance Series). The Cu Kα radiation (λ = 1.54178 Å) was generated at 40 kV, and the electric current was adjusted to 40 mA. Spectra were measured at a rate of 0.05 min^–1^ and the angle of diffraction was varied from 5° to 80°.

#### 2.3.6 Thermal Properties Determination

TGA analysis was performed using Shimadzu TG-60WS equipment. Nanofibrous mat samples were heated from 20 °C up to 650 °C at a rate of 10 °C min^–1^ under air. DSC (TA Instruments, Q10) was also utilized using the ramp method in a range of 10–300 °C at 10 °C min^–1^ under N_2_ atmosphere (20 mLmin^–1^).

## 3 Results and Discussion

### 3.1 Electrical Conductivity, Surface Tension, Viscosity, pH, Density of Solutions

Protein solutions are conductive. The solution must have an electrical conductivity that establishes the repulsion of charge to overcome the surface tension of the droplet for nanofibrous structure formation.^35^ Low conductivity can cause a decrease in the charge density on the jet surface of the spinning solution. Electrical conductivity is proportional to the ion concentration of the solution, which increases when there are more ions.^36^ A 40% FV extract alone had an electrical conductivity of 84.60 ± 1.92 μS cm^–1^. Increasing the FV against the zein proportion caused a decrease in electrical conductivity (Table 1). Similarly, Göksen et al.^37^ reported that the electrical conductivity of a zein solution decreased from 523.2 μS cm^–1^ to 417.6 μS cm^–1^ with essential oil addition. pH could affect the conductivity of the biopolymer solution and, therefore, the electrostatic repulsion force. During the electrohydrodynamic processing, pH affects the molecular conformation of the nanofibrous mats.^38^ Higher pH might reduce the protonation of functional groups, leading to stronger intermolecular and intramolecular interactions (Figure 1) and causing higher crystallinity.

**Figure 1:**
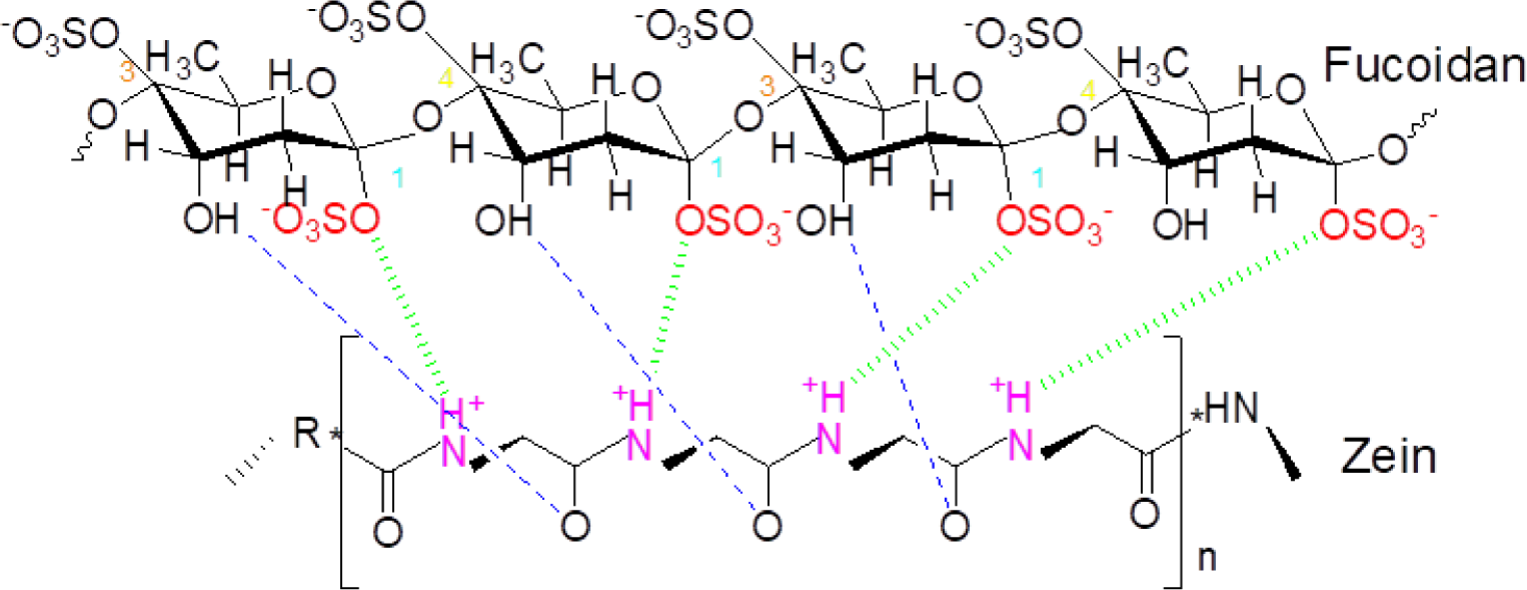
Fucoidan-zein intermolecular and intramolecular interactions.

**Table 1:** FV:Z 3:1 and FV:Z 4:1, and FV Extract Solution Properties

	pH	Density (gml^–1^)	Electrical Conductivity (μscm^–1^)	Viscosity (mPa.s)	Surface Tension (mN(m^2^)^−1^)
**FV:Z 3:1**	4.2	1.26 ±0 .005	79.70 ± 1.08	261.40 ± 7.15	23.08 ± 0.36
**FV:Z 4:1**	4.5	1.24 ± 0	78.20 ± 1.54	209.61 ± 8.21	22.92 ± 0.23
**FV extract solution**	5.1	-	84.60 ± 1.92	175.29 ± 10.53	26.69 ± 0.09

It has been stated that adsorption behavior changes above pH 4.^39^ At acidic conditions, the −NH_2_ of the zein binds with H+ ions, producing the −NH^3+^. Then an interaction occurs between the −NH^3+^ and the anion form of the bioactive group of FV due to the electrostatic attractions between active sites. This interaction increases the density because the addition of H+ ions adds mass to the solution. At higher pH (4.2), the removal efficiency of the sorbent beads decreased due to the breakup of OH^–^ ions with the active sites of the adsorbents. It brings a negative charge to the bioactive ions above this pH, and the repulsion forces between the negatively charged bioactive compounds and the surface layer of the zein, making the solution less cohesive.^39^ Electrostatic interactions form a crucial pathway in bioactive compounds from Fucus/zein coacervation due to their sensitivities to pH, ionic strength, and biopolymer ratios.^40^

With more zein against FV extract (for FV:Z 3:1), chains are protonated, and the solution’s viscosity is higher.^36^ The consistency coefficient (*k*), flow behavior index (*n*), and other parameters for FV:Z 3:1 and FV:Z 4:1 are presented in Table 1. Shear stress (τ) increased nonlinearly with increasing shear rate (γ), indicating shear-thinning behavior and a deviation from Newtonian flow. The solutions exhibited pseudoplastic fluid behavior with yield stress. The consistency coefficient (*k*) was between 0.027 and 0.038 at these pH ranges. Solutions had shear-thinning behavior (flow behavior index *n* < 0.9). Solutions showed variation in rheological properties, with *n* ranging from 0.80 to 0.90, similar to the study by Bernaerts et al.^41^

The hydrophilic nature of fucoidan allows for interactions with zein molecules possessing complementary hydrophobic regions. Therefore, the stability and bioactivity improved with the formation of intermolecular associations. Zhang et al.^42^ successfully utilized fucoidan to stabilize zein in nanocomplex form at different pH, temperature, and salt concentration conditions, and obtained a good delivery effect. They reported that electrostatic attraction and H bonding were the main driving forces for their nanocombination.

### 3.2 Morphological Structure

Changes in the interaction of biopolymers in a solvent may form a distinct organization in the formed polymer chains, which influences the properties of nanofibrous mats.^43^ A 70/30 ethanol/water solvent resulted in nanofibrous mats with an average diameter of 547.5 ± 264.4 and 420.0 ± 123.9 for FV:Z 3:1 and FV:Z 4:1, respectively (Figure 2). The morphology of electrospun nanofibrous mats can be affected by electrospinning parameters, polymer concentration, surface tension, or acidic conditions. It was found that the 4:1 FV:Z solution is ideal to obtain thinner fibers. For FV:Z 3:1, there were fewer beads compared to those in FV:Z 4:1. This could be explained by the higher viscosity and electrical conductivity increasing the viscoelastic force, which resulted in more entanglements between polymer chains, producing fewer beads.^15^ Furthermore, higher density favors the formation of thicker fibers, where fibers are deposited in a higher packing density on the collector, creating thicker nanofibrous mats.^44^ Standard deviations indicated that a high-acid environment leads to a nonuniform aggregation pattern, resulting in a highly polydisperse system of electrospun mats.

**Figure 2:**
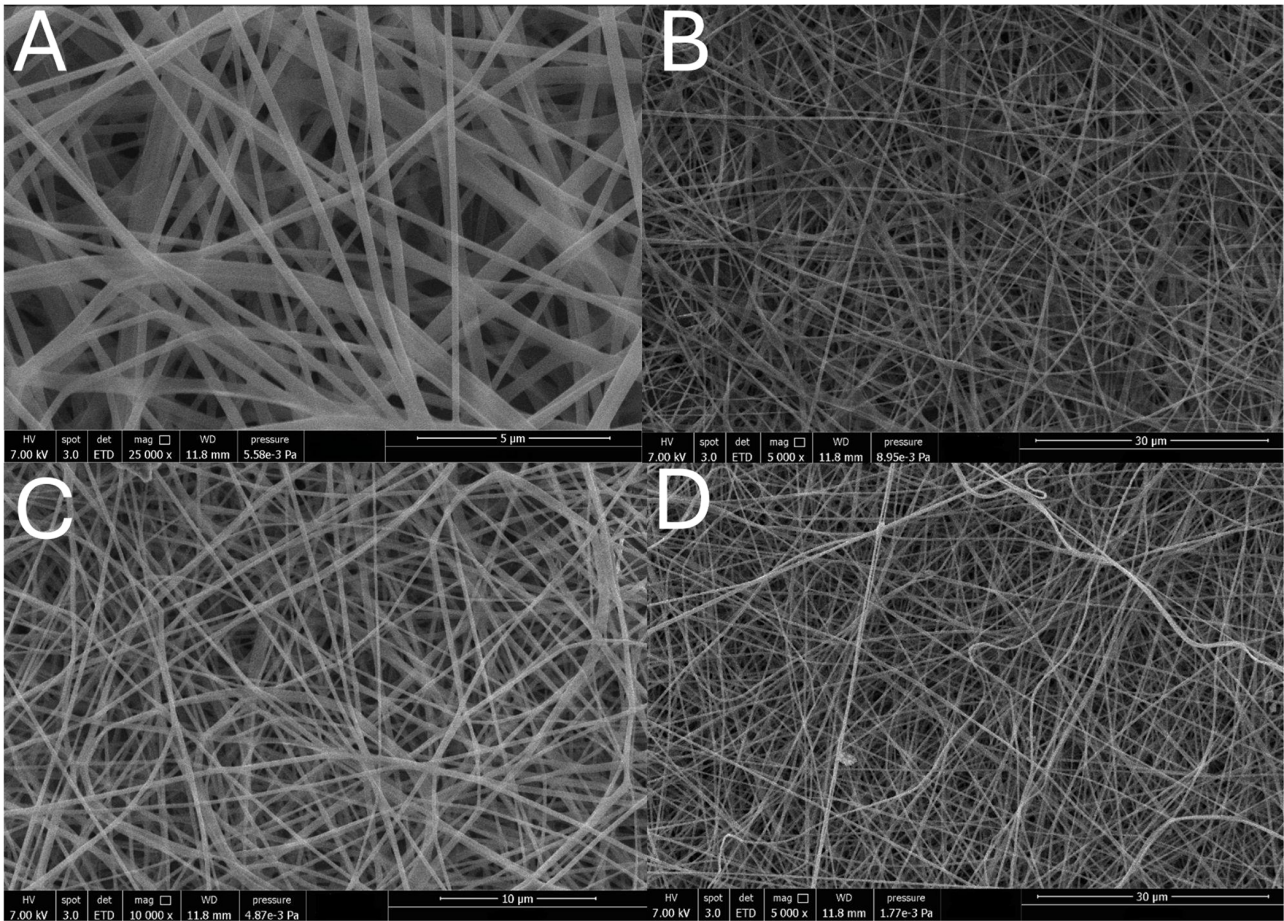
FV:Z 3:1 (A, B) and FV:Z 4:1 (C, D) electrospun nanofibrous mats. Magnifications: A: 25,000×, B and D: 5000×, C: 10,000×.

The tubular shape of fibers was attributed to the unfolding of Fucus/zein proteins under acidic conditions, as described in the solution pH results. Here, it could be concluded that Fucus/zein chains folded because of the solvent effect. Unfolding of zein results in a higher density of surface-active residues, which increases inter- and intramolecular junctions, changing the interfacial properties such as the surface tension of FV:Z solutions.^40^ Therefore, intermolecular interactions with H bonding could cause smooth and defect-free nanofibrous mats.

### 3.3 Surface Properties and Gibbs Energy

BET analysis was performed to measure the specific surface area and porosity properties of the nanofibrous mats. Porosity and surface area are important in the reaction mechanism, recovery, and molecular interaction processes. The BET specific surface area of FV:Z nanofibrous mats obtained from zein-doped Fucus is shown in Table 2.

**Table 2:** Specific Surface Area, Total Pore Volume, Average Pore Diameter of Electrospinning Solutions and Nanofibrous Mats, and Thermodynamic Parameters of the Samples

Parameter	FV:Z 4:1 Solution	FV:Z 3:1 Mats	FV:Z 4:1 Mats
**Specific surface area** **(m** ^**2**^ **g** ^**–1**^)	95.23	100.09	105.06
**Total pore volume** **(cm** ^**3**^ **g** ^**–1**^)	0.017	0.020	0.024
**Average pore diameter (nm)**	1.518	1.713	1.748
Δ ***G*** (kJmol^–1^)	-	–48.96	–55.78
Δ ***H*** (kJmol^–1^)	-	–110.26	–115.21
Δ ***S*** **(kJ(mol.K)** ^–1^)	-	–0.28	–0.33

Compared to FV:Z electrospun nanofibrous mats, the electrospinning solution (FV:Z 4:1) showed different results. An increase in the specific surface area, average pore size, and pore volume occurred in nanofibrous mats formed by the interaction of Fucus and zein under the electrical field. This may be due to the movement of a larger amount of Fucus particles on the zein biopolymer during electrospinning.^45^

The increase in surface is attributed to the presence of dipole–dipole interactions that strengthen and stabilize the weak network in the polymer (cationic and/or anionic)/bioactive matrix connected by London (weak van der Waals) attractive forces and inter/intramolecular H bonding.^46^ Porosity increases the contact time of zein/Fucus bioactive components (substituents S1 and S2) and improves the interactions and diffusion of molecules, functionalization of structural compounds, and transfer of the reaction through the nanofibrous matrix, which increases the physicochemical and mechanical efficiency.^47^

The interaction between zein and FV polyphenol compounds was characterized by negative Δ*S* values. This exothermic value indicated that the number of effective collisions between Fucus and active sites in zein increased (the number of reactants per unit increased) and positively contributed to the formation of more stable phases. The higher negative enthalpy value (Δ*H*) for 4:1 FV:Z indicated that the active end groups surrounding the polyphenols and zein formed a more ordered structure when the interaction occurred, and the surface interactions of certain binding sites were consistent. In addition, the negative Δ*G* value indicated that the binding of zein to the polyphenols was thermodynamically spontaneous.^48^ The more negative the Δ*G* value, the greater the degree of spontaneity of that reaction, and the less negative the Δ*G* value is attributed to the lesser spontaneity of that reaction.^49^ Higher Δ*G* values with negative signs indicate chemical interactions. In general, the Δ*G* for physical interaction is between zero and −20 kJmol^–1^, and for chemical interactions, it is between −40 and −500 kJ mol^–1^. Also, negative values of Δ*G* indicate that the energy of the bond between zein and bioactive compounds is stronger.^47^ Wan der Waals and H bonding played an important role in the interactions between zein and FV polyphenol compounds, which were determined to be less than 0. Zein–Fucus polyphenol interactions allow prediction of the behavior of polyphenol self-assembled zein polymers.

Overall, the mechanical stability of the FV:Z nanofibrous mat structure increased at a 4:1 ratio, and the reaction mechanism progressed homogeneously, with the bonds gained stability, which improved the efficiency. These results were in accordance with other characterization studies described in other sections.

### 3.4 Total Antioxidant Capacity and Radical Scavenging Activity

Studies have shown that FV extracts can scavenge free radicals. Fucoidan polysaccharides lead to maintaining the structure and integrity of nanomaterials during storage. Their hydrophilicity preserves the properties of bioactives, ensuring nanomaterial stability.^21^ The radical scavenging activity of Fucus polyphenols is based on reactions and also depends on the amount and location of the −OH groups and their phenolic structure (Figure 3).

**Figure 3:**
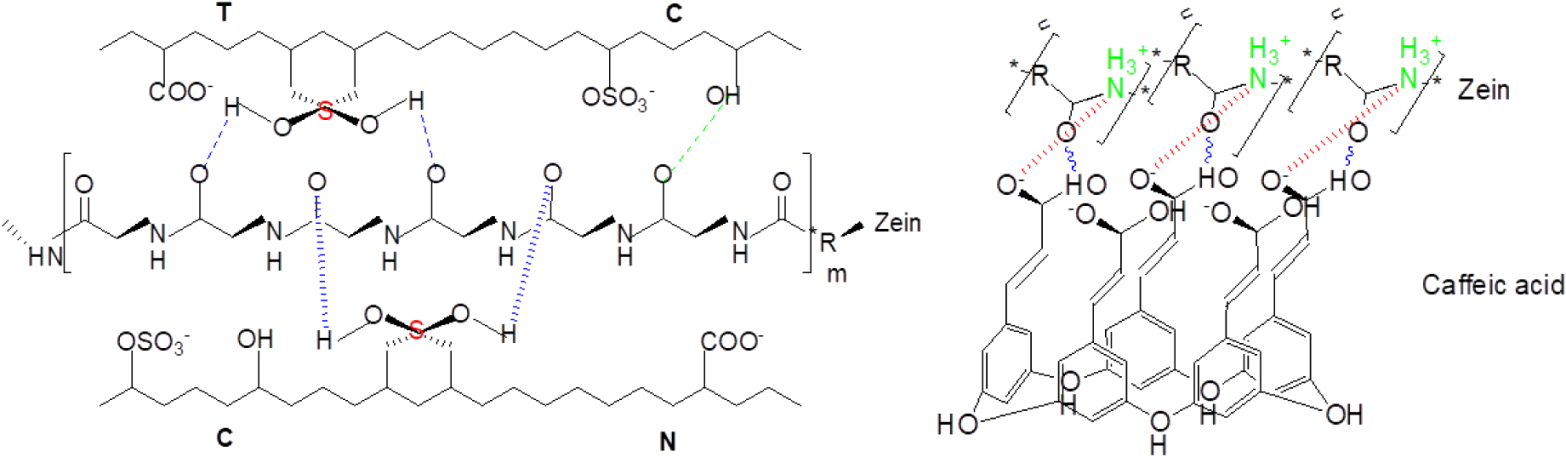
Zein-Fucus bioactive compound interactions and an example of zein-FV caffeic acid interaction.

The reported data on the antioxidant activity in the microalgae extracts are generally measured by DPPH and ABTS assays. Their colorimetric methods were established to reveal the relationship between the antioxidant activity of the extract and the electron donor capacity. In this method, absorbance increases as a result of the color change when the radical reaction H is scavenged by antioxidants through the donation of H· to form intramolecular H bonding. Antioxidant activities and their calibration curves of FV:Z 3:1 and FV:Z 4:1 nanofibrous mats are shown in Tables 3 and 4 and Figure 4, respectively.

**Table 3:** ABTS and DPPH Results of FV:Z 3:1 Electrospun Nanofibrous Mats

Concentration	Inhibition% (DPPH)	Reduction% (ABTS)	IC50 μg ml^–1^(DPPH)	IC50 μg ml^–1^(ABTS)	Total IC50 (DPPH)	Total IC50 (ABTS)
2 μg ml^–1^	9.93 ± 1.15	4.40 ± 0.75	0.95	0.924	47.05	46.37
5 μg ml^–1^	39.44 ± 0.64	18.97 ± 0.46	2.05	1.369		
10 μg ml^–1^	73.82 ± 0.84	42.09 ± 0.52	7.05	6.369		
18 μg ml^–1^	83.13 ± 0.55	67.18 ± 0.46	15.05	14.369		
25 μg ml^–1^	90.97 ± 1.07	85.68 ± 0.45	22.05	21.369		

**Table 4:** ABTS and DPPH Results of FV:Z 4:1 Electrospun Nanofibrous Mats

Concentration	Inhibition% (DPPH)	Reduction% (ABTS)	IC50 μg ml^–1^(DPPH)	IC50 μg ml^–1^ (ABTS)	Total IC50 (DPPH)	Total IC50 (ABTS)
2 μg ml^–1^	10.21 ± 0.36	5.04 ± 0.27	0.99	0.845	47.01	46.90
5 μg ml^–1^	39.38 ± 0.42	17.65 ± 0.71	2.01	1.9035		
10 μg ml^–1^	73.40 ± 0.64	41.80 ± 0.64	7.01	6.9035		
18 μg ml^–1^	83.61 ± 0.32	65.68 ± 0.66	15.01	14.9035		
25 μg ml^–1^	90.28 ± 0.87	85.60 ± 0.75	22.01	21.9035		

**Figure 4:**
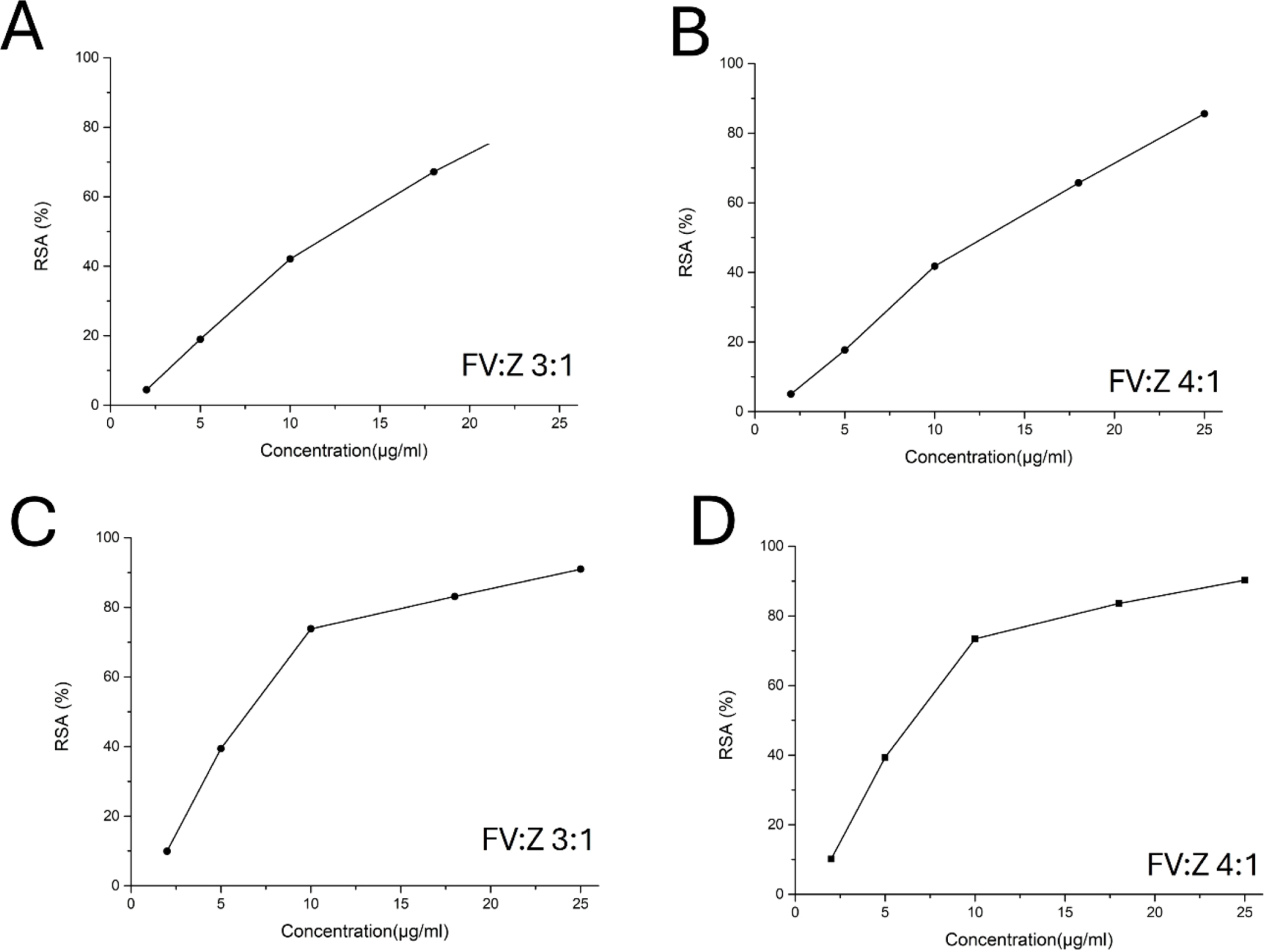
ABTS (A, B) and DPPH (C, D) calibration curves of nanofibrous mats.

Antioxidant activity is expressed by the IC50 value, meaning that the higher the antioxidant activity, the lower the IC50 value. The greater content of polyphenols is likely to be related to the lower IC50 activity of nanofibrous mats. The IC50 radical-scavenging effects of DPPH were noticed by antiradical power with inhibition treatment values for a TPC (phenolic acids, flavonoids, and flavonols). The TPC total IC50 values were between 46.37 and 47.05 μgml^–1^ for nanofibrous mats, which are higher than previous reports for FV.^50,51^ Nova et al.^52^ also noticed rich polyphenolic activities in FV extracts. ABTS radical scavenging activity was increased to >90% when polyphenol concentration was 25.0 μgml^–1^. The electron chain reaction in phenolic substances provides the transfer of H-molecules to free radicals, which then become radicals. The results of the present study indicated that the ABTS activity may be due to the presence of carbonyl and −OH group vibrations in the FV.

FV:Z 3:1 and FV:Z 4:1 nanofibrous mats showed the highest DPPH radical scavenging activity of 90.97 ± 1.07% and 90.28 ± 0.87, respectively, at a concentration of 25 μg ml^–1^ in methanolic solvent. The total antioxidant capacity of ABTS was reached at 85.68 ± 0.45% and 85.60 ± 0.75% with the maximum tested concentration. The concentration that provided 22.05% and 22.01% of DPPH inhibition (IC50) corresponded to a TE of >90 μmol Trolox g^–1^, while for the ABTS assay, the reduction (IC50) was attained at a tested concentration of 21.37% and 21.90%, representing a TE of >85 μmol Trolox g^–1^. To understand the interaction between antioxidant activity and total phenolic compounds, a Pearson correlation was calculated for each phenolic acid. ABTS (*R* ^2^, 0.995 and *R* ^2^, 0.9959) and DPPH activity (R^2^, 0.9295 and R^2^, 0.9290) showed a positive correlation with polyphenol compounds. The Pearson correlation supports that polyphenols are the primary antioxidant compounds present in nanofibrous mats. Yu et al.^12^ stated that oligomerization of polyphenol components appears to be important for DPPH and ABTS scavenging assays. They investigated phlorotannins from FV extract using Peleg’s correlation between polyphenol compounds and antioxidant capacity.

Results indicated that nanofibrous mats containing fucoidan polysaccharides and fucoxanthin could lead to a greater protective effect by enhanced radical neutralization and chelation of metal ions. Nanofibrous mats also improved the stability and antioxidant activity, retaining the nanoencapsulated FV components’ biological activity at ambient temperatures.

### 3.5 FTIR Results

Studies revealed that complete information on nanomaterial’s characteristics is important before their use, and FTIR is helpful for molecular-level characterization.^53^ Figure 5 shows the FTIR spectra of pure zein and FV extract, their mixed FV:Z 3:1 and 4:1 solutions, and produced nanofibrous mats. As is known, characteristic peaks of zein at 1630, 1520, and 1240 cm^–1^ correspond to amides I, II, and III, respectively. Pure zein follows: 1652 cm^–1^ Amide I band (C=O stretching vibration), 1516 cm^–1^ Amide II band (N–H bending vibration), 1246 cm^–1^ Amide III band (C–N and N–H in-plane bending vibration), and 1019 cm^–1^ is related to the C–O group. The peak at 1253 cm^–1^ could represent sulfonic acids of fucoidan polysaccharides (Figure 5; KR) and the amine group of zein (Figure 5; DS) binding.^16,54^

**Figure 5:**
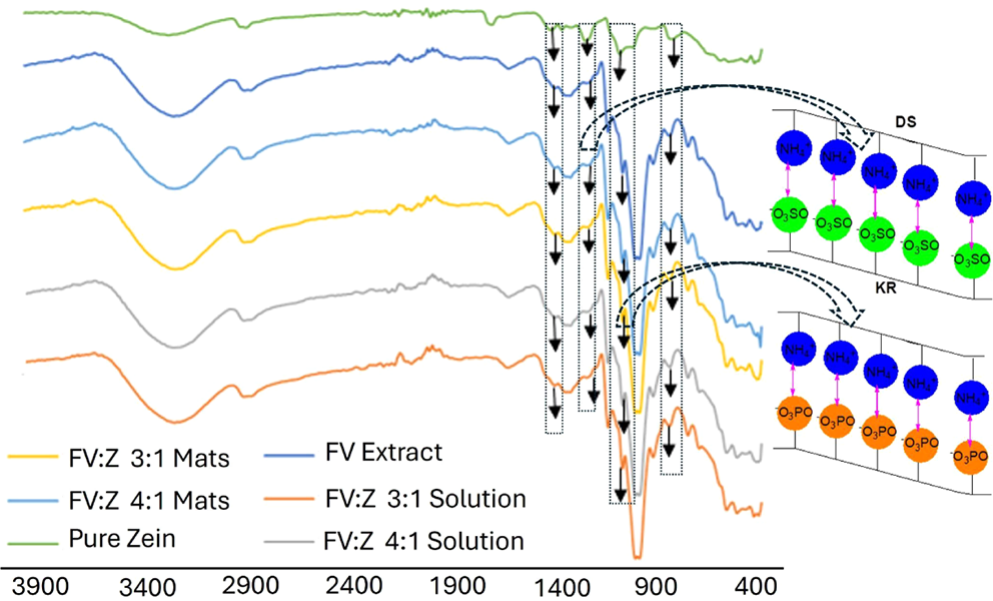
FTIR spectra of pure zein, FV extract, FV:Z 3:1 and 4:1 solutions, and produced nanofibrous mats after normalization of the ordinate.

The FTIR spectrum of FV extract shows sharp and halo peaks at 1600–1400 cm^–1^ and 1300–1900 cm^–1^, representing the proteins and carbohydrates (related to O–H stretching, symmetric and asymmetric stretching), respectively.^16^

A strong absorption band at 2925 cm^–1^ (C–H bonding vibration) could be related to the characteristics of lipids. Changes between 2400 and 2000 cm^–1^ could be related to the decomposition of calcium minerals in algae.^55^ Because, after interaction with the solvent (water and ethanol), Ca decomposes to Ca­(OH)_2_ and CaO_2_, which may oxidize to CO_2_ and CO under the applied electrical voltage. Youhong et al.^56^ stated that high-voltage breakdown frequency causes pyrolysis. Overall, this region may show the decomposition of CaO_2_ and CaOH_2_ with electrical force during electrospinning. So, CO formation could be attributed to the decomposition of CaO_2_ minerals with electrical force and pyrolysis (Figure 6A,C). On the other hand, Wang et al.^57^ examined polystyrene-*Chlamydomonas reinhardtii* chlorophyll π–π (stacking, anion, alkyl) interactions in this region of the spectra. According to those findings, Zein π–π stacking and ionic interaction with Fucoxanthin pigment may also have occurred (Figure 6B,C).

**Figure 6:**
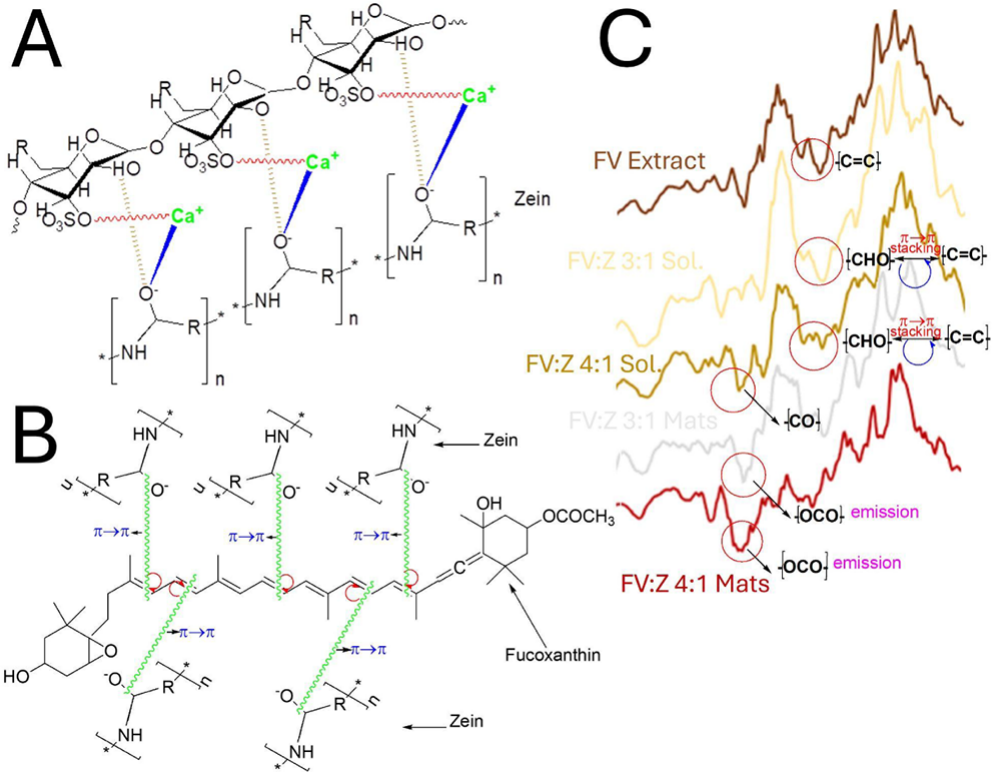
FV compounds-zein chemical bond formation mechanisms (A, B) and their representation in the FTIR 2400–1900 region with closer magnification (C).

Amide I and II specific bands were detected in all samples containing the FV extract. In these regions, after zein participation, shoulders with little shifts were reported, which could be the result of the formation of physical cross-linking between Fucus compounds and zein proteins. Here, the positively charged nitrogen atom in zein is stabilized by the negatively charged polar groups from Fucus. After electrospinning, Amide I peaks were shifted from 1626 cm^–1^ to 1628–1632 cm^–1^ due to protein unfolding and changes in secondary structure or H bonds between fucoidan–zein compounds.^16^ When they are dissolved in a solvent, protein structures change because of denaturation and unfolding under a high electric field. Electric fields elongate the proteins and alter their conformation. Ethanol evaporation favors the assembly of monomeric units into β-sheets and random coils in an environment of increasing hydrophilicity, promoting the development of intermolecular interactions to form small oligomers.^16^ This trend could be explained by changes in band start–end differences, which were presented in Table 2.

The band in the range of 1084–1023 cm^–1^ represents C–O–H stretching vibrations of carboxylic acids or P–O–C bonds of phosphate groups.^58,59^ Widening in the bands at 1010–1000 cm^–1^ in nanofibrous mats (Figure 7) compared to the FV extract shows that van der Waals intermolecular and π–π interactions occur between zein carboxyl groups and FV S–H groups. Unfolded zein can aggregate by hydrophobic and SH–SS interactions, but the reactivity depends on pH. The difference in widths between FV:Z 3:1 and FV:Z 4:1 could be attributed to this trend.

**Figure 7:**
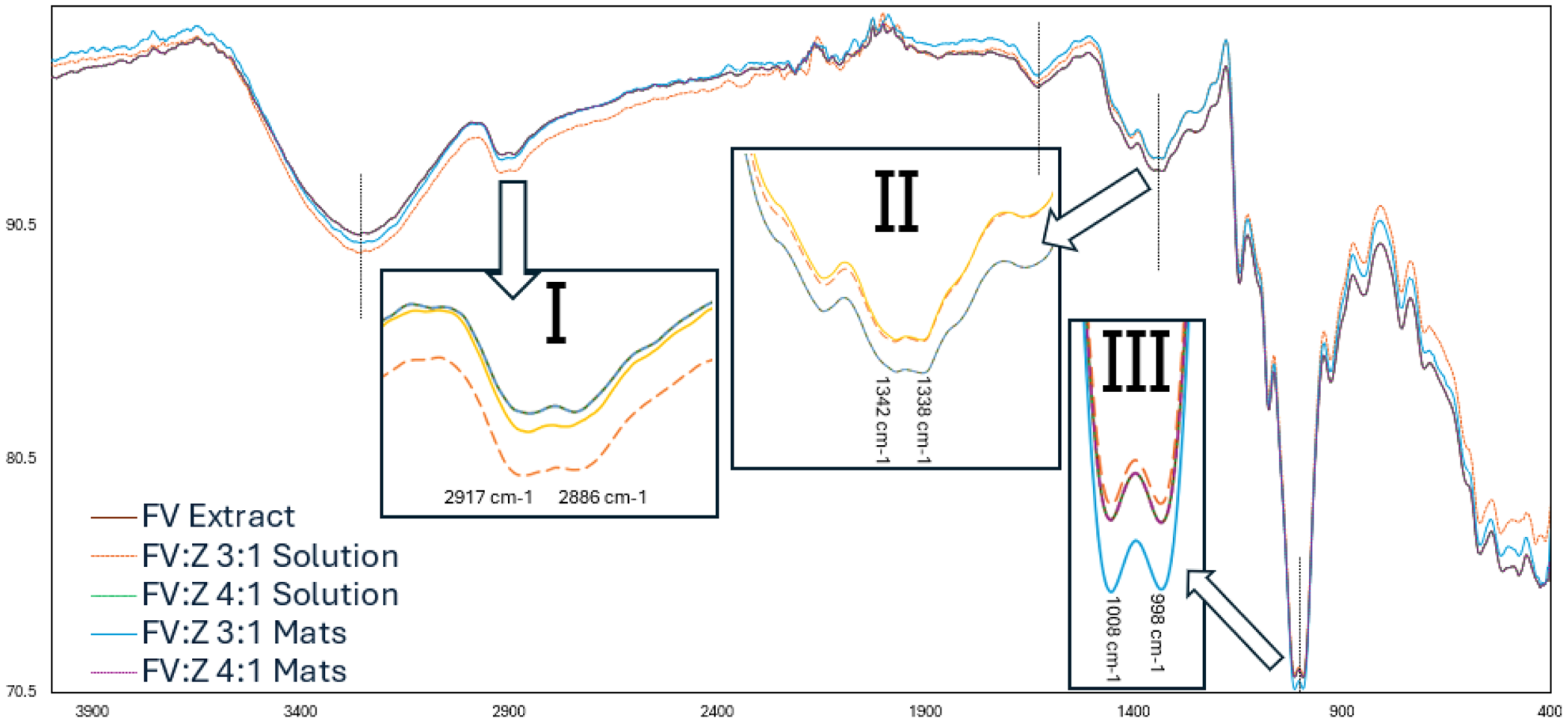
FTIR spectra of samples with important peak positions, partially magnified and indicated by arrows.

Moreover, in some previous studies, peaks at 1083^–1^ and 1034 cm^–1^ were attributed to β-mannuronic acid and α-L-guluronic acid^11^ or reflect the glycosidic bonds (C–O–C).^58,60^ After the addition of zein, peaks were shifted (Table 5) because of interactions.^11,16,58,59,61^ Finally, shoulders around 750–700 cm^–1^ are related to the aromatic C=H out-of-plane bending.

**Table 5:** Peak Positions and Related Bonds for Samples

Sample Peak Position (cm^–1^)			
FV Extract	FV:Z 3:1 Mats	FV:Z 4:1 Mats	Peak Assignment
3253.42	3257.91	3258.65	Stretching vibrations of the −OH group
2912.81	2909.36	2910.47^I^ ^a^	ϑ_as_(CH2) asymmetric stretching of hydrocarbons
2202.37	2208.41	2208.96	δ_s_(−CO−) deformation cationic groups (X^2+^) from fucoxanthin, ϑ_as_(−OCO−) stretches of fucoidan polysaccharides
1911.73	1916.75	1914.52	
1626.39	1628.50	1632.84	Amide from protein ϑ_s_(C–N) stretching and δ(N–H) bending vibrations
1433.54	1439.21	1437.07	Methylene and δ(CH_2_) scissoring vibrations of ester lipids groups
1562.24	1561.37	1560.85	δ trans (−C =C−) carotene and – δ(NH) nucleic acids groups
1344.86	1346.19	1348.44^II^ ^a^	ϑ_s_(CH_3_) symmetric stretches bend protein and lipids
1256.18	1254.07	1253.86	δ(=CH_2_) deformation from lipids, ϑ_s_(−O=S=O) symmetric stretches vibration of sulfate ester
1238.67	1240.10	1245.28	(−CH_2_) ring bend from β-carotene, phosphodiester, phospholipids
1139.53	1145.27	1144.66	δ_as_(C–O–C) asymmetric bend from polysaccharides and fucoxanthin
1116.64	1118.92	1120.01	ϑ_s_(−P–O) symmetric stretches from nucleic acids
1080.27	1079.48	1079.94	ϑ_as_(=P–O) and ϑ(C–O–P) or ϑ(P–H) asymmetric and symmetric stretches from phospholipid and nucleic acids
1027.96	1030.53	1031.17	ϑ(−S–O) stretching vibrations of polysaccharides carbohydrates
1002.60	1004.07	1005.51^III^ ^a^	δ_s_(−S=O of sulfoxides) symmetric bending carbohydrates from fucoxanthin
919.13	926.54	928.08	ϑ(−C–C−) stretching vibration (angular) of β-and ϑ_s_(P–O–P) symmetric stretches vibration of polyphosphate groups
889.67	885.01	886.56	ϑ_s_(−CH) of β- galactopyranose residues and, δ(−C–S–O) vibration of phospholipid ester groups
836.09 801.08	842.42 799.05	842.95 794.63	ϑ_s_ cis(-HC=CH_2_) and ϑ_as_(−CH_2_) symmetric and asymmetric vibrations of molecules in TAGs (triacylglycerides)
750.81 707.50	754.94 702.68	755.20 701.89	(−C–H) out of plane vibrations of α-linkages and δ_S_(−C–O–C) bending vibrations of α-glycosidic linkages
676.99	670.27	668.03	-ϑ(OH) deformation vibration of the hydroxyl group of bioactive fragments
606.49	610.81	612.27	–(C–C) bonds chains of hydrocarbons

a I; II; III: Figure 7.

As a common structure, β-sheet is an H bond formed between the carbonyl oxygen (−C=O) and amide hydrogen (−CONH−) of amino acids through peptide bond linkage.^62^ β-sheet structural changes in nanofibrous mats are the reason for cross-linked interpenetrated Fucus-zein network.

### 3.6 Phenolic Content of Nanofibrous Mats

Algae show various total phenolic content due to factors such as geographical cultivation area, season, and environmental variations. Extraction conditions also influence the total phenolic content. Polymeric forms of phloroglucinol, phlorotannins, can be found in brown seaweed, which play an important role in protection against UV radiation and defense.^12^

Machu et al.^63^ examined the effects of ethanol, methanol, aqueous methanol, and water solvent on phenolic contents and antioxidant capacity in brown algae extracts and stated that amounts can vary according to the used aprotic solvent. High antioxidant capacity and high phenolic content determination in the current study were attributed to the efficiency of the applied methods, which is given in Section 3.3.

Lutein and apigenin were identified in nanofibrous mats, which are an important source in microalgae species.^64^ Lutein is an antioxidant flavonoid, and its presence in Fucus-based mats has been proven, as shown in the HPLC graphs (Figure 8). Fernandes et al.^65^ detected transluteins in the two microalgae Specie *C. sorokiniana* (831.18 ± 1.18 ng mg^–1^) and *S. bijuga* (526.40 ± 4.40 ng mg^–1^), respectively. In this study, 1105 ± 0.05 and 1110.00 ± 0.01 ng mg^–1^ lutein were detected in FV 3:1 and FV 4:1 mats, showing a high value of translutein (Table 6). The production of lutein is significant in some microalgae species and is considered a potential source of commercial output for this pigment.^66^ As confirmed by the graphic abstract photos, the liquids exhibited different colors depending on the sample during ABTS and DPPH determination, which is attributed to their lutein content.

**Figure 8:**
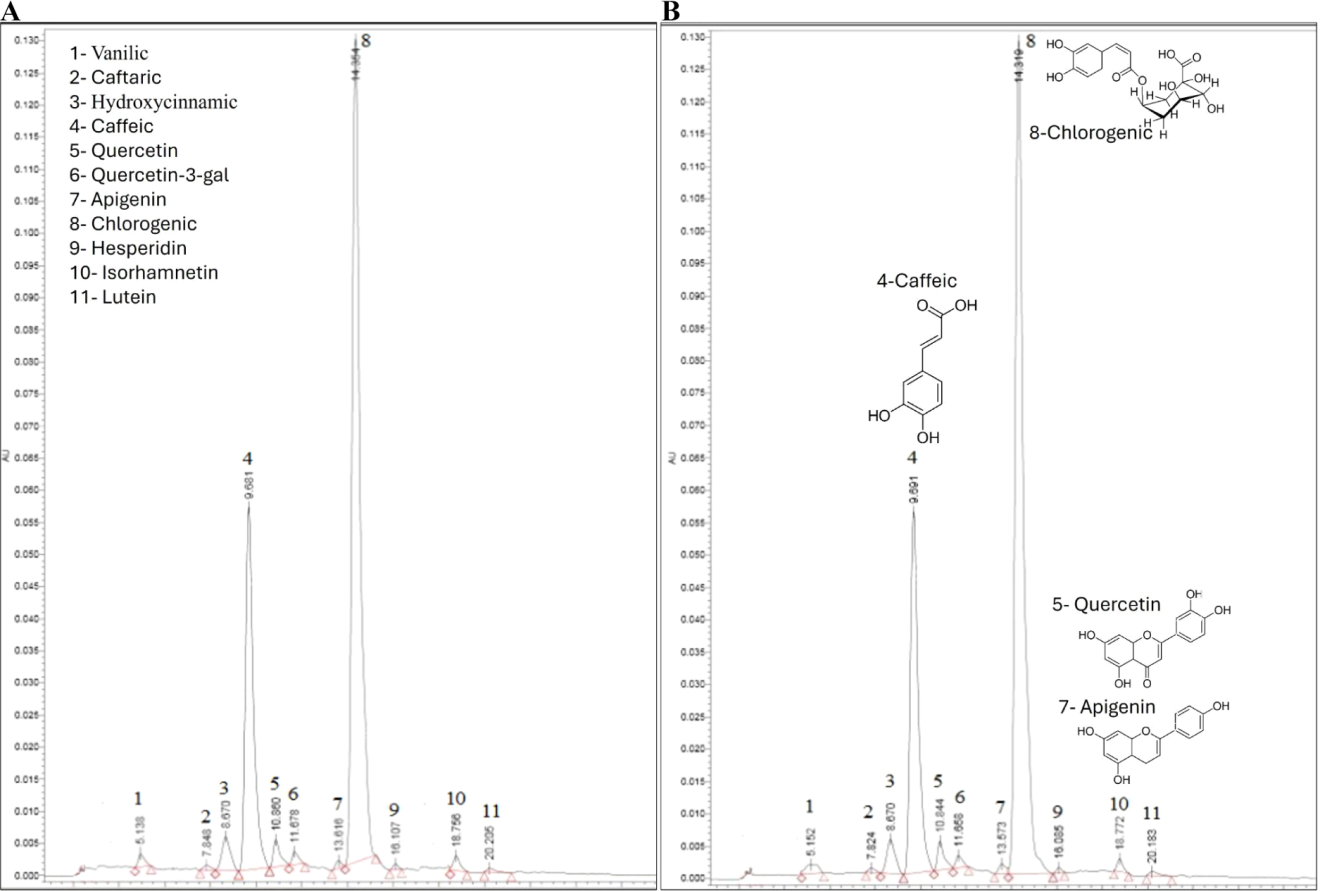
Phenolic acids in FV:Z 3:1 (A) and 4:1 (B) identified by HPLC, with retention times.

**Table 6:** Determined Polyphenolic Compound Amounts (μg mg^–1^) in FV Electrospun Mats Identified by HPLC and Comparison with Previous Studies

Compound (ngmg^–1^)	Current Study^a^	Sánchez-Bonet el al.^67^	Rossi et al.^1^	Catarino et al.^3^
Vanillic	16.01	6.86	7.67	10.86
Caftaric	9.26	ND	ND	3.25
Hydroxycinnamic	21.5	10.72	14.2	14.73
Caffeic	16.23	3.95	11.39	13.41
Quercetin	19.8	<LOD	7.8	9.37
Quercetin-3-gal	8.66	<LOD	<LOD	ND
Apigenin	9.31	ND	2.27	5.42
Chlorogenic	7.18	13.28	9.25	8.46
Hesperidin	7.46	ND	ND	2.39
Isorhamnetin	11.9	ND	ND	ND
Lutein	10.5	ND	3.5	4.08

a Average values of FV:Z 3:1 and FV:Z 4:1 samples.

Isorhamnetin and Hesperidin were also found, which, to the best of my knowledge, were not seen in previous studies . Scaglioni et al. (2019) stated that chlorogenic acid predominated in the phenolic extract from *Nannochloropsis* sp. Similarly, chlorogenic acid had the highest amount of phenolic content in nanofibrous mats after Isorhamnetin and Hesperidin in the current study. In their study, they identified seven different phenolic acids compared to 11 in the nanofibrous mats. Thus, it is attributed that a variety of phenolic compounds could show a synergic effect, which may also influence the nanofibrous mats’ antimycotoxigenic action.

It has been stated that Quercetin and Quercetin 3-Gal compounds exhibit potential antioxidant activity as well as anti-inflammatory and anticancer properties.^68^ Since these compounds were also detected in samples (1.98–1.99 and 8.66–8.46 ngmg^–1^), it can be concluded that nanofibrous mats could be used for antioxidant activities.

Vanillic and caffeic acids were identified as reported previously.^69–71^ Tannic acid was also found (data not shown) in samples but was below the limits. Tannic acid specifically regulated metabolite production without directly impacting microalgal cellular physiological properties. Zou et al.^72^ stated that polyphenols could be biodegraded by microbial metabolism potential. Therefore, the determined gallic acid in electrospun mats could be a metabolite of the tannic acid degradative pathway.

### 3.7 XRD Results

The peaks in the XRD pattern were successfully determined and are presented in Figure 9. Results show differences from some other recent studies in terms of peak intensities. For instance, Madany et al.^58^ could not observe distinct peaks in the XRD patterns. These differences could be attributed to the working current range (milliampere parameter) of the XRD equipment. Therefore, the working current range seems to be an important parameter for nanofibrous mats.

**Figure 9:**
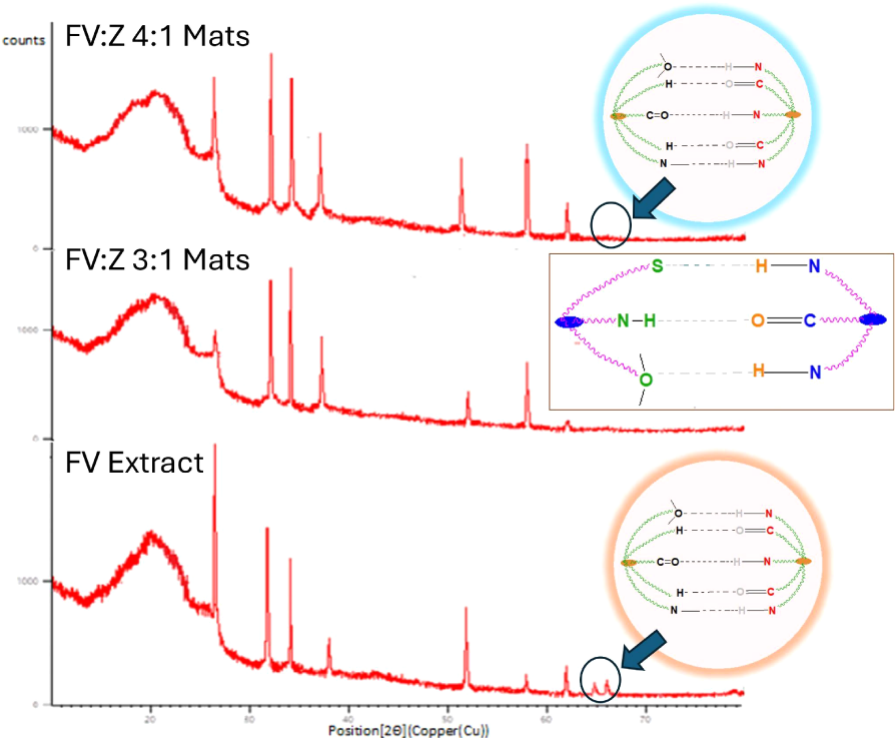
XRD results of the samples.

Samples showed semicrystalline behavior, having both crystalline and amorphous regions. Madany et al.^58^ stated that ulvan has an indistinct backbone and branching structure. This heterogeneous composition results in a disordered conformational structure and the existence of amorphous regions. Repeated aldobiouronic units could clarify the crystalline region. Since the pH of the FV:Z 4:1 solution was lower than FV:Z 3:1, higher intensity peaks in FV:Z 3:1 may be attributed to the crystalline effect of acidic phenolic compounds.^73^

As seen in Figure 9 changes (shifting, peak intensity) in amorphous or crystalline regions and disappearing peaks at (65°–66°) could be attributed to H-bonding between secondary −OH groups and the zein cationic -NH^2^ group, which contribute to stabilization.^74^ The highly branched bioactive groups and negatively charged SO_4_ molecules of fucoidan also contributed to the stability. Fucoidan’s high negative charge density and ability to interact with glycoproteins inhibit molecular aggregation.^21^

A broad peak between 5 and 25° is attributed to the semicrystalline structure of nanofibrous mats. However, at around 22°, the nanofibrous mats’ peak had less intensity and seemed more splayed than the FV extract peak, probably due to rapid evaporation of ethanol and water in the Fucus-zein solvent. Here, polymer chains have insufficient time to arrange themselves in an ordered structure and solidify in an unordered form.^15^

22° exhibited a noncrystalline structure for the FV extract. The diffractogram shows that the nanofibrous mats have a crystalline form, represented via diffraction peaks at 19° and 23°. After production of the mats, the reflection of FV extract peaks showed relatively lower intensity and position changes (Table 7). 26.99° peak was higher in FV extract and FV40 compared to FV30 (FV effect). The 38.14° peak positions shifted to the left and showed higher intensities in nanofibrous mats (zein effect). Forghani et al.^75^ stated that ∼2θ = 33° may correspond to gallic acid compounds. The FV extract peak position at 57.95° moved to 58.03° and stabilized for mats, but FV:Z 3:1 had lower intensity than FV:Z 4:1. 64.86° and 65.97° showed intensities for FV extract, but there were no peaks in nanofibrous mats.

**Table 7:** Important Peak Positions and Corresponding Relative Intensities for FV extract, FV:Z 3:1, and FV:Z 4:1 Samples

Peak Position [°2θ]			Relative Intensity (%)			FWHM Left [°2θ]		
FV Extract	FV:Z 3:1	FV:Z 4:1	FV Extract	FV:Z 3:1	FV:Z 4:1	FV Extract	FV:Z 3:1	FV:Z 4:1
19.68	19.90	20.01	73.86	70.09	73.64	0.11	0.10	0.10
26.99	26.99	27.00	102.41	74.32	115.06	0.10	0.10	0.10
32.08	32.08	32.46	128.45	120.03	124.18	0.10	0.10	0.10
34.03	34.07	34.09	124.85	121.99	128.16	0.10	0.10	0.10
38.14	38.03	37.86	79.39	87.53	91.21	0.12	0.11	0.10
51.97	52.04	52.09	89.94	79.86	89.24	0.10	0.10	0.10
57.95	58.03	58.03	73.01	85.56	88.36	0.10	0.10	0.10
61.98	61.87	62.09	73.82	65.47	69.47	0.10	0.12	0.13
64.86	-	-	62.07	-	-	0.11	0.40	0.40
65.97	-	-	62.61	-	-	0.12	0.41	0.52

Overall, it can be concluded that the cationic and anionic end groups of zein interact with the more reactive substitute C6 OH groups, causing changes in peak intensity and disappearance, especially in the C2 regions, compared to the FV extract.^75^ Changes in intensities of XRD peaks show that molecular aggregations were affected, which could be related to the −H bonds and van der Waals molecular interactions between zein and phenolic acids and, as a result, rearrangements in crystalline phases.

### 3.8 Calorimetry and Thermogravimetric Results

The DSC results of FV extract, FV:Z 3:1, and FV:Z 4:1 nanofibrous mats are presented in Figure 10A. The DSC thermogram of FV extract showed endothermic peaks with midpoint transition temperatures at 71.9 °C, 93.6 °C, and 157.5 °C. These peaks could be related to denaturation temperatures. Mosayebi et al.^15^ reported thermal transition values at 85 °C for Spirulina protein isolate. Pereira et al.^73^ obtained denaturation temperature values of 96.7 and 107 °C for Spirulina protein isolate and protein concentrate, respectively.

**Figure 10:**
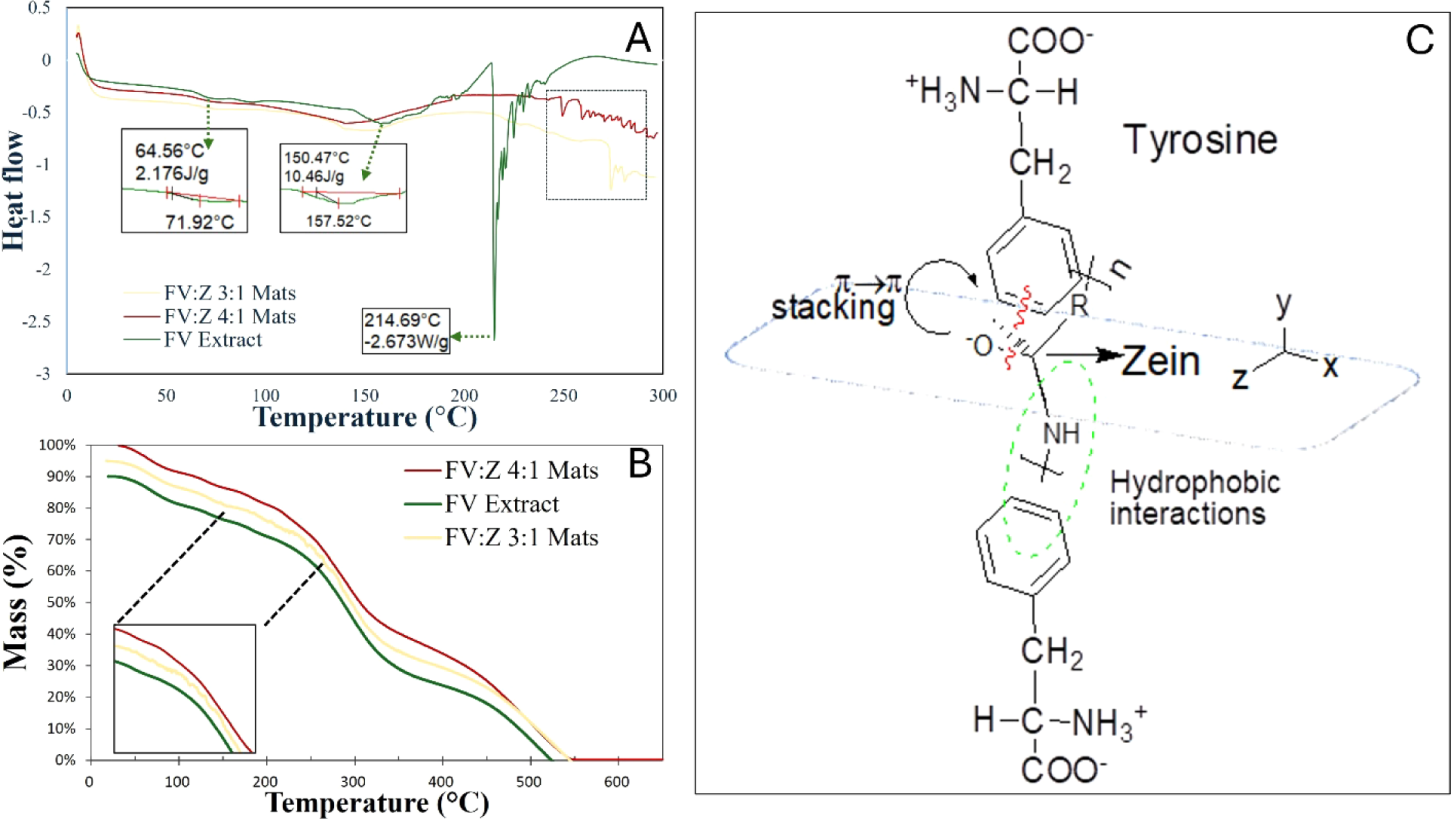
DSC (A) and TGA (B) thermograms of samples and interaction mechanisms that improve thermal behavior (C).

DSC curves showed a large number of endothermic peaks after 250 °C for nanofibers, which were not seen for the FV extract after this temperature. This may correspond to a polymer blend of FV extract-zein thermal degradation temperatures. Gonçalves et al.^43^ stated that polycaprolactone electrospun nanofibers have endothermic peaks when microalgae peptides are integrated. Similarly, in a study by Danesin et al.,^76^ multiple endotherms were observed in DSC thermograms of nanofibers after peptide incorporation.

In a study by Chao et al.,^77^ the main polyphenol ingredient of chlorogenic acid had a peak around 210 °C, and after forming a physical mixture or complex, it peak shifted to lower or higher temperatures. In the current study, the FV:Z 3:1 and FV:Z 4:1 mat decomposition curves were different from the FV extract, which could be attributed to the increasing interaction ability of cationic end groups of FV molecules with the zein hydrophobic groups and helix bonds (Figure 10C). Applied voltage affects protein structure by reducing helical domains and destroying intraprotein H bonds, which is consistent with the XRD results.^78^ Compared to the FV extract (214.7 °C), mats showed distinct bands in decomposition temperature with shifting, attributed to the electrohydrodynamic process that results in increased segmental mobility of the FV extract-zein polymer mixture.^15^

TGA analysis leads to observing the thermal degradation mechanism, determining the amount of moisture and volatile compounds, weight loss, and thermal breakdown of the mats.^79^ The first weight loss step at 50–200 °C shows the evaporation of water and volatile compounds (Figure 10B). Golmakani et al.^16^ stated that fucoidan had the first level of physically absorbed water evaporation around ∼70 °C. Here, around a similar temperature, FV:Z 4:1 had less moisture loss than other samples, which might be explained by FV extract/zein interactions via H bonds. This leads to the reduction of the number of free −OH groups that give the hydrophilic character to electrospun mats, and thus a decrease in moisture uptake with increasing FV extract proportion.^80^

The second stage, at the range of 225–400 °C, was the main thermal degradation zone of the samples. Golmakani et al.^16^ indicated that between 220– and 280 °C, fucoidan started to lose chemisorbed water and its molecular structure. Fast loss of mass between 250 and 350 °C is due to the degradation of the polymer chains.^81^ Higher thermal stability with less weight loss was observed in nanofibrous mats compared to FV extract due to the interaction between bioactives in FV and zein, along with crystallinity changes.^75^ Here, splittings in denaturation temperatures in FV:Z 3:1 nanofibrous mats could be attributed to a complex process including protein chain rupture and peptide bond breakage.^15^

Improvement in thermal performance of mats is due to the van der Waals and H bonds between the polyphenols, and the zein may provide more stability at high temperatures. Results suggest that this enhancement might be due to bioactive components being composed of phenolic acids, providing a mass contribution to the electrospun mats.

The last stage of weight loss (400–550 °C) was due to decomposition of thermally resistant compounds of FV extract, and then byproduct formation.^15^ Pouralkhas et al.^82^ indicated that after 500 °C, fucoidan curves underwent a random breakage of glycosidic linkages. The remaining mass after 500 °C was changed into ash, which may represent fucoidan minerals such as PO_4_, CO_3_, and SO_4_.^82^ However, in the third step, weight loss in mats was delayed to higher temperatures compared with the FV extract. Nanoscale dimensions allowed much more contact area, and therefore, more resistance was observed with increasing temperature.^83^

Overall, the electrospinning process enhanced the thermostability of FV extract, leading to increased hygroscopicity and greater polymer interaction.

## 4 Conclusion

Smooth nanofibrous mats were formed using only an ethanol:water solvent mixture from FV extract and zein. Increasing the FV extract ratio to 4:1 resulted in a 23.3% reduction in the diameter of the nanofibrous mats; correspondingly, the specific surface area of the material and porosity increased. It was also observed in BET analyses and Gibbs energy results that fucus-zein complexes appear to have spontaneous electrostatic interactions and covalent and noncovalent bond formations without pH and temperature intervention. The FV extract-based nanofibrous mats were a source of polyphenols and displayed high antioxidant activities. ABTS and DPPH assays showed that at the lowest sample concentration (2 μgml^–1^), FV:Z 4:1 had better results, while when the concentration dose increased up to 25 μgml^–1^, the opposite case occurred. The higher active substrate to inhibit free radicals (<IC50) indicated that the scavenging activity of the nanoencapsulated extract has contributed to chemical stability. FTIR spectra indicate C–O–P, P–O–P bonds, SO_4_^–2^ groups, and the sugar cycle of glycosidic linkages with changed intensities between fucoidan/zein nanostructures after nanofibrous mat formation. Moreover, microalgae pyrolysis under an electrical field with applied voltage showed CO_2_ and CO decomposition peak formations between 2400 and 2000 cm^–1^. Zein formed aggregates due to conformational transformations of monomeric units that affected the α-helix structures. FV extract’s negatively charged molecules affected zein’s aggregation by forming electrostatic complexes through interactions with positively charged regions on zein molecules. Cationic and anionic Fucus end groups of peptide chains showed triple-helix conformer properties after combination with zein in nanoform. DSC results were compatible with this observation. It can be concluded that zein contributes to the thermal stability of the nanofibers, since at different FV:Z ratios, the mat’s degradation temperatures varied. The 250–300 °C transition zone in the mats was found to be the interface between adjacent H-bonded molecular sheets, which was consistent with the XRD data. Using FTIR, TGA, and DSC analyses, multiple interactions between the Fucus:zein chains were elucidated. These analyses led to the determination of various possible reaction routes and interaction mechanisms. It can be concluded that the FV extract-zein mixture could be utilized as a shell material for nanoencapsulation of bioactives and has potential for use in food formulations, which is a limitation of the current study. Therefore, the developed nanomaterials with high antioxidant activity can be used in food products in the future.

## Supplementary Material



## Notes

Rheological parameters of electrospinning solutions and FTIR band distance differences in samples (PDF)
