# Supplementary material for: Physicochemical, Structural, Molecular, and Thermal Characterization of Fucus vesiculosus Extract-Based Nanofibrous Mats
Source: ACS Omega. 2026 Jan 12;11(3):4239–53. doi: 10.1021/acsomega.5c09347 (PMC12854499; doi:10.1021/acsomega.5c09347)
Supplement: Supplementary file 1 [file ao5c09347_si_001.pdf]

Supporting Information

Physicochemical, Structural, Molecular, and Thermal Characterization of *Fucus vesiculosus* Extract-Based Nanofibrous Mats

Fatih Bildik*

Istanbul Technical University, Faculty of Chemical and Metallurgical Engineering, Department of Food Engineering, 34469, Istanbul, Türkiye

*Corresponding Author: bildikfatih@itu.edu.tr

TABLE S1. Rheological parameters of FV:Z 4:1 and FV:Z 3:1 electrospinning solutions according to Ostwald de Waele and Bingham models.

Sample	Ostwald de Waele Model			Bingham Model		
	K<br>Consistency Coefficient<br>Pascal s (Pa·s^n)	n<br>Flow Behavior Index	r	τ0<br>Yield Stress Pascal (Pa)	η_p<br>Plastic Viscosity Pascal s (Pa·s)	r
FV:Z 3:1(I)	0.02782	0.8031	0.9904	0.1847	0.01826	0.9999
FV:Z 3:1(II)	0.03047	0.8455	0.9976	0.1729	0.01548	0.9999
FV:Z 4:1(I)	0.03894	0.9013	0.9917	0.1960	0.01414	0.9991
FV:Z 4:1(II)	0.03518	0.8414	0.9985	0.1674	0.02282	0.9994

TABLE S2. Band starting-ending distance differences (centimeters) in FV extract, solutions, and nanofibrous mats.

Average band starting and ending transmittance	FV Extract	FV:Z 3:1 Solution	FV:Z 4:1 Solution	FV:Z 3:1 Mat	FV:Z 4:1 Mat
3700-3100	0.156	0.170	0.338	0.188	0.321
1720-1560	0.108	0.114	0.195	0.124	0.220
1530-1280	0.251	0.266	0.340	0.272	0.386
1230-950	0.239	0.249	0.561	0.256	0.612
